# Transcriptome Analyses Reveal the Molecular Response of Juvenile Greater Amberjack (*Seriola dumerili*) to Marine Heatwaves

**DOI:** 10.3390/ani15131871

**Published:** 2025-06-24

**Authors:** Yali Tian, Liancheng Li, Hongzhao Long, Dongying Zhang, Chen Wang, Ruijuan Hao, Hang Li, Xiaoying Ru, Qiuxia Deng, Qin Hu, Yang Huang, Chunhua Zhu

**Affiliations:** 1 Southern Marine Science and Engineering Guangdong Laboratory (Zhanjiang), Zhanjiang 524006, China; tianyali0301@163.com (Y.T.); a18348379766@163.com (L.L.); longhz2023@163.com (H.L.); zhangdy@zjblab.com (D.Z.); wangc@zjblab.com (C.W.); lih@zjblab.com (H.L.); ruxiaoying@zjblab.com (X.R.); dengqiuxia@zjblab.com (Q.D.); huq@zjblab.com (Q.H.); zjouhy@126.com (Y.H.); 2 Fisheries College, Guangdong Ocean University, Zhanjiang 524088, China; 3 Guangdong Research Center on Reproductive Control and Breeding Technology of Indigenous Valuable Fish Species, Guangdong Ocean University, Zhanjiang 524088, China; 4 Guangdong Provincial Engineering Laboratory for Mariculture Organism Breeding, Zhanjiang 524088, China

**Keywords:** marine heatwaves, oxidative stress, immune function, energy metabolism, *Seriola dumerili*

## Abstract

Marine heatwaves (MHWs) have recently become more frequent, intense, and prolonged, posing significant threats to marine life and fisheries. In this study, transcriptomic analysis was employed to investigate the genes and pathways in *Seriola dumerili* that respond to MHW-induced stress at 28 °C (T28) and 32 °C (T32), using 24 °C (T24) as the control. Transcriptome sequencing revealed that 17 differentially expressed genes (DEGs) belonging to the heat shock protein (HSP) families—HSP30, HSP40, HSP70, and HSP90—were significantly upregulated under short-lasting MHW stress in the T24-4d vs. T32-4d comparison. Additionally, genes related to oxidative stress (e.g., protein disulfide isomerase family A member 6 [*pdia6*]), immune responses (e.g., interferon regulatory factor 5 [*irf5*]), and energy metabolism (e.g., hexokinase-1 [*hk1*]) were also identified. Enrichment analysis of DEGs in the T24-4d vs. T32-4d group revealed that *S. dumerili* exhibited adaptive responses to MHWs through the upregulation of HSPs and the activation of antioxidant, energy metabolism, and immune response pathways. However, in the T24-13d vs. T32-13d group, DEGs associated with these pathways were either not significantly expressed or were downregulated. These findings indicate that *S. dumerili* is unable to sustain its adaptive responses under repeated, intense MHW exposure, resulting in the disorder of its antioxidant defense system, immune suppression, and metabolic dysfunction. This study provides valuable insights into the molecular responses of *S. dumerili* to MHWs and supports the selection for thermal resistance in this species.

## Simple Summary

Marine heatwaves (MHWs) impact the sustainability of marine life and fisheries, including commercially important species such as *Seriola dumerili*. However, little is known about how this species responds to the stress of MHWs at the molecular level. In this study, we examined the gene expression changes and biological processes involved in the response of *S. dumerili* to short-lasting and repeatedly occurring MHWs. These findings provide a scientific foundation for understanding the adaptive mechanisms of *S. dumerili* to MHWs conditions.

## 1. Introduction

Against the backdrop of human-accelerated global warming, ocean warming has garnered widespread attention [1]. In aquatic environments, temperature is a key abiotic factor that influences fish growth, reproduction, behavior, and overall health [2]. Within a certain range, increased temperatures can promote metabolic activity and enhance growth in aquatic organisms [3]. A fluctuation of just 1 °C in water temperature can alter a fish’s metabolic rate by approximately 10% [4]. In response to temperature-induced stress, fish activate various physiological mechanisms and cellular responses to mitigate damage [5], including adjustments in energy production processes [6], the regulation of oxidative stress, and modulation of immune responses [7]. Organisms can withstand intense thermal stress within their species’ thermal limits by initiating short-term stress responses, such as cellular stress mechanisms that protect and repair macromolecular systems while minimizing non-essential metabolic activities [8]. However, exposure to high temperatures outside the optimal range adversely affects fish, triggering a cascade of stress responses that can result in slowed growth and weakened immunity [9]. Extreme temperatures compromise the immune function of aquatic organisms, increasing their vulnerability to disease. This not only intensifies the challenges in fish farming but also leads to significant economic losses in the aquaculture industry due to persistent pathophysiological disorders and high mortality rates in certain species [10]. As a result, fisheries researchers have increasingly focused on promoting the healthy growth of aquatic organisms in the context of global warming [11].

Marine heatwaves (MHWs), characterized by anomalous increases in seawater temperatures, are a significant manifestation of extreme events driven by global warming [12]. These events can span vast oceanic regions and persist for durations ranging from days to months, with climate change projected to further increase their frequency and intensity [13]. Over the past century, more than 50% of the world’s oceans have experienced an increase in the annual number of MHW days [14], and MHWs with substantial ecological impacts have become increasingly frequent in recent years [15]. MHWs have been associated with mass mortality events in marine invertebrates and benthic fish [16], widespread coral bleaching and mortality [17], and the rapid decline in kelp forests and the associated seaweed communities [18]. Critically, regional fisheries and aquaculture industries are becoming increasingly vulnerable to these extreme thermal events [19]. The increasing occurrence of MHWs represents a major stressor, contributing to increased disease incidence, higher mortality rates, and reduced productivity in marine organisms [20]. Although the vulnerability of marine ecosystems and fisheries to MHW events is well acknowledged, research into their specific impacts remains limited [21]. In our previous study, we investigated the effects of MHWs on physiological indicators in juvenile *Seriola dumerili*, demonstrating that the species responds to MHW-induced stress by modulating the activity of the enzymes related to energy metabolism, antioxidant defense, and immune function [22]. Nevertheless, the molecular response of fish to MHWs remains unstudied.

The greater amberjack *S. dumerili* is a marine species widely distributed in subtropical waters, with populations found in regions such as China, southern Japan, northern India, and Australia [22]. *S. dumerili* is highly valued by consumers for its high-quality meat and nutritional content, making it a promising species for global aquaculture and offering significant potential for industry development [23]. Previous research has shown that *S. dumerili* exhibits a circumglobal distribution and can tolerate a broad temperature range, from 15 °C to 27 °C [24]. However, the molecular mechanisms underlying its response to MHWs remain largely unknown. In this study, we conducted a transcriptomic analysis of liver tissue from *S. dumerili* exposed to MHW conditions to investigate its temperature acclimatization mechanisms. These findings are expected to support the development of high-temperature-tolerant *S. dumerili* strains for aquaculture.

## 2. Materials and Methods

### 2.1. Experimental Design

**Figure 1:**
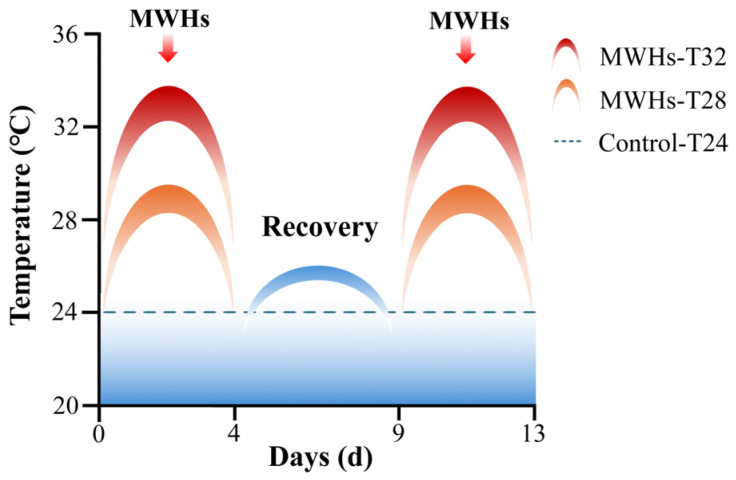
Experimental design of short-lasting and repeatedly occurring MHWs. MHWs-T32: Severe MHW treatment at 32 °C; MHWs-T28: Moderate MHW treatment at 28 °C; Control-T24: Average seawater temperature control at 24 °C.

A total of 120 juvenile *S. dumerili* (average body weight: 128.80 ± 28.17 g; age: 7 months) from farmed populations were used in this experiment. The fish were randomly assigned into three groups of 40 individuals each and housed in an indoor aquaculture facility. The culture conditions were maintained with constant salinity (29 ‰), dissolved oxygen (9.5 ± 0.5 mg/L), and pH (8.0 ± 0.3). An MHW experiment was conducted following the protocol described by Tian et al. [22] (Figure 1). The survival rate of *S. dumerili* decreased to 65% at 32 °C (T32), while 100% survival was observed at both 24 °C (T24) and 28 °C (T28) [22]. The experimental temperatures were set at 24 °C (T24, control), 28 °C (T28), and 32 °C (T32), with exposure durations of 4 days (short-lasting MHWs) and 13 days (repeatedly occurring MHWs). All the groups were first acclimated at the baseline temperature of 24 °C for 7 days before being exposed to the gradually increased experimental temperatures including 28 °C and 32 °C. On both day 4 and day 13 of exposure, six individuals were randomly selected from each treatment group. The fish were anesthetized using eugenol, and liver tissues were immediately dissected and stored at −80 °C for transcriptomic analysis.

### 2.2. Transcriptome Analysis

Six individuals from each group (T24, T28, T32) at two time points (4 days and 13 days) were used for transcriptome analysis, with every two individuals pooled to form one sample. This resulted in a total of 18 transcriptomes, with three biological replicates per group. Total RNA was extracted from each individual using the TRIzol reagent kit (Thermo Fisher, Sunnyvale, CA, USA). Equal amounts of RNA from every two individuals were combined to create one sequencing sample. The quantity and purity of total RNA were assessed using the Bioanalyzer 2100 and the RNA 6000 Nano LabChip Kit (Agilent Technologies, Santa Clara, CA, USA; Cat. No. 5067-1511). Only RNA samples with an RNA integrity number (RIN) greater than 7.0 were used for library construction. Sequencing was performed on the Illumina Novaseq™ 6000 platform (Illumina, San Diego, CA, USA). To obtain high-quality clean reads, raw sequencing reads were further filtered using Cutadapt (version 1.9) to remove reads containing adapter sequences, polyA or polyG tails, more than 5% unknown nucleotides (N), and over 20% low-quality bases (Q-value ≤ 20). The quality of the clean reads was then assessed using FastQC 1.0.0, evaluating metrics such as Q20, Q30, and GC content. Clean reads were aligned to the *S. dumerili* reference genome [25] using the HISAT2 package. The gene expression abundance was quantified with StringTie (version 2.1.6). DEGs were identified using DESeq2, applying a false discovery rate (FDR) cutoff of <0.05 and an absolute log_2_fold change ≥ 1. Heatmaps of DEGs were generated using the OmicStudio tools (https://www.omicstudio.cn/tool) (accessed on 20 January 2025) [26]. Subsequently, DEGs were annotated using the Gene Ontology (GO) database (http://www.geneontology.org/) (accessed on 10 July 2023) and mapped to the Kyoto Encyclopedia of Genes and Genomes (KEGG) database (http://www.genome.jp/kegg/) (accessed on 10 July 2023) for pathway analysis.

### 2.3. Quantitative Real-Time Reverse Transcription Polymerase Chain Reaction Validation

To validate the transcriptome analysis, nine DEGs potentially involved in the response to heat stress—including those related to HSPs, oxidative stress, and immune response—were selected. The primers for these genes were designed using Primer 5.0 software (Table S1). Total RNA was extracted from *S. dumerili* liver samples using the TRIzol reagent (TransGen Biotech, Beijing, China) according to the manufacturer’s instructions. Complementary DNA (cDNA) was synthesized, and genomic DNA was removed using the *TransScript*^®^ Uni All-in-One First-Strand cDNA Synthesis SuperMix for qPCR kit (TransGen Biotech, Beijing, China). A quantitative real-time reverse transcription polymerase chain reaction (qRT-PCR) was conducted on a CFX Opus 96 Real-Time PCR Detection System (Bio-Rad Laboratories, Hercules, CA, USA) with the use of the *PerfectStart*^®^ Green qPCR SuperMix kit, following the manufacturer’s protocol (TransGen Biotech, Beijing, China). The qRT-PCR amplification employed pre-denaturation at 94 °C for 30 s followed by 40 cycles of 94 °C for 5 s and then 60 °C for 30 s. Relative gene expression levels were calculated using the comparative Ct method (2^−ΔCt^), with β-actin serving as the internal reference gene [27,28].

## 3. Results

### 3.1. Transcriptome Sequencing Quality

A total of 872.11 million clean reads were obtained from the liver transcriptomes under MHWs (Table 1). The Q30 values ranged from 95.03% to 98.01%, indicating high sequencing quality. The alignment rates of clean reads to the reference genome ranged from 93.90% to 97.11%, confirming the suitability of the data for downstream analyses.

### 3.2. DEG Identification and Analysis

In the comparison between T24-4d vs. T28-4d, 39 DEGs were identified, including 17 upregulated and 22 downregulated genes. The T24-13d vs. T28-13d group showed 164 DEGs, with 62 upregulated and 102 downregulated. In contrast, the T24-4d vs. T32-4d comparison revealed a much larger number of DEGs—2390 in total—including 1379 upregulated and 1011 downregulated genes. Similarly, T24-13d vs. T32-13d had 1775 DEGs, consisting of 577 upregulated and 1198 downregulated genes (Figure S1). The substantially higher number of DEGs in T24 vs. T32 compared with T24 vs. T28 indicates that the T32 condition imposed a stronger heat stress challenge on *S. dumerili* during MHWs. Therefore, subsequent analyses focused on the T24 vs. T32 comparisons to investigate the impact of MHWs on *S. dumerili*.

**Figure 2:**
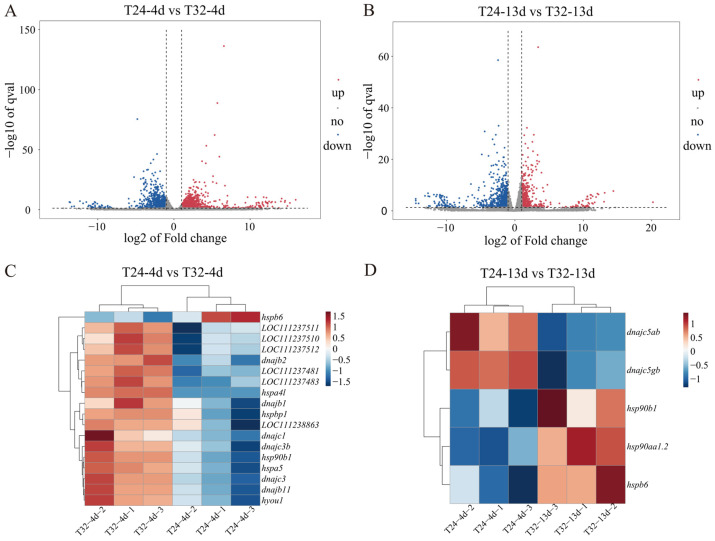
Visual analysis of DEGs in the T24 vs. T32 group. (**A**,**B**) Volcano plots of DEGs for T24-4d vs. T32-4d and T24-13d vs. T32-13d, respectively; (**C**,**D**) heatmaps showing the expression of HSP genes for T24-4d vs. T32-4d and T24-13d vs. T32-13d, respectively. The heatmaps show the FPKM of DEGs (|log_2_fold change| ≥ 1 and *p* < 0.05), which are transformed and normalized. Hierarchal cluster was performed with the method of complete linkage. Red indicates upregulated DEGs, and blue indicates downregulated DEGs in the comparable groups.

The overall distribution of DEGs is illustrated by volcano plots in Figure 2A,B. In the T24 vs. T32 group under short-lasting MHWs, 18 HSP genes were significantly differentially expressed, including 17 upregulated genes such as Hsp30 (LOC111237481, LOC111237483, LOC111237511, LOC111237510, and LOC111237512), Hsp90 (*hsp90b1*), and Hsp40 (*dnajc3*, *dnajb11*, *dnajb2*, *dnajb1*, *dnajc3b*, and *dnajc1*). Other upregulated HSP genes included Hsp70 (*hspa5*, *hspa4l*, LOC111238863, and *hyou1*) and HSPB (*hspbp1*). Only one HSP gene, sHsp (*hspb6*), was downregulated (Figure 2C). During repeatedly occurring MHWs, five HSP genes showed significant expression changes: three were upregulated [Hsp90 (*hsp90aa1.2* and *hsp90b1*) and sHsp (*hspb6*)], while Hsp40 genes (*dnajc5ab* and *dnajc5gb*) were downregulated (Figure 2D). In addition, genes associated with oxidative stress (such as protein disulfide isomerase family A member 6 (*pdia6*), endoplasmic reticulum oxidoreductase 1 alpha (*ero1a*), and prolyl 4-hydroxylase beta polypeptide (*p4hb*)), immune responses (including interferon regulatory factors 5 (*irf5*) and 8 (*irf8*)), and energy metabolism (such as hexokinase-1 (*hk1*) and hexokinase-2-like (*hk2*)) were also identified (Tables S2 and S3).

### 3.3. GO Enrichment Analysis

**Figure 3:**
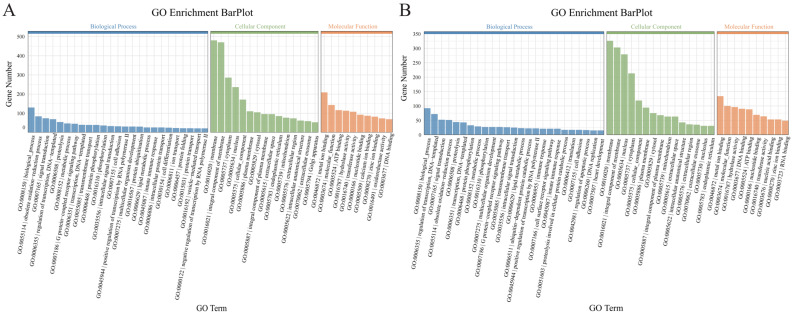
GO enrichment analysis of DEGs in the T24 vs. T32 group. (**A**,**B**) Bar graphs showing the top 25, top 15, and top 10 terms with the highest number of differentially expressed genes annotated in the molecular function, cellular component, and biological process categories, respectively, for T24-4d vs. T32-4d and T24-13d vs. T32-13d.

The DEGs from the T24-4d vs. T32-4d and T24-13d vs. T32-13d groups were annotated into three main categories: biological processes, cellular components, and molecular functions (Tables S4 and S5). Within the biological process category, most DEGs were involved in oxidation–reduction processes, signal transduction, and metabolic processes (Figure 3A,B). For cellular components, the majority of DEGs were associated with cell membranes, cellular parts, and organelles (Figure 3A,B). Regarding molecular function, most DEGs in both groups were related to binding and catalytic activity (Figure 3A,B).

### 3.4. KEGG Pathway Enrichment Analysis

**Figure 4:**
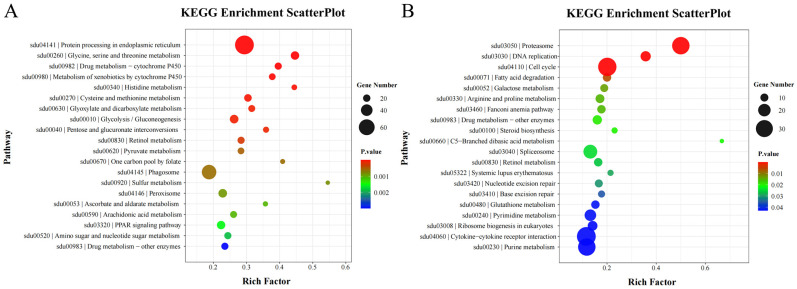
KEGG enrichment analysis of DEGs in the T24 vs. T32 group. (**A**,**B**) Bubble plots showing the top 20 most significantly enriched KEGG pathways at T24-4d vs. T32-4d and T24-13d vs. T32-13d, respectively.

A total of 235 and 212 pathways were identified in the T24-4d vs. T32-4d and T24-13d vs. T32-13d groups, respectively (Tables S6 and S7; Figure 4A,B). Several pathways related to immune defense (e.g., NOD-like receptor signaling and Toll-like receptor signaling), antioxidation (e.g., protein processing in the endoplasmic reticulum (ER), peroxisome, and apoptosis), and energy metabolism (e.g., glycolysis/gluconeogenesis, fatty acid degradation, and histidine metabolism) were enriched in both groups (Tables S6 and S7). In immune-related pathways, most DEGs were upregulated in the T24-4d vs. T32-4d group but downregulated in the T24-13d vs. T32-13d group (Table S8). Meanwhile, DEGs in energy metabolism pathways were significantly regulated only in the T24-4d vs. T32-4d group (Table S9).

### 3.5. Validation of Transcriptome Data Using qRT-PCR

**Figure 5:**
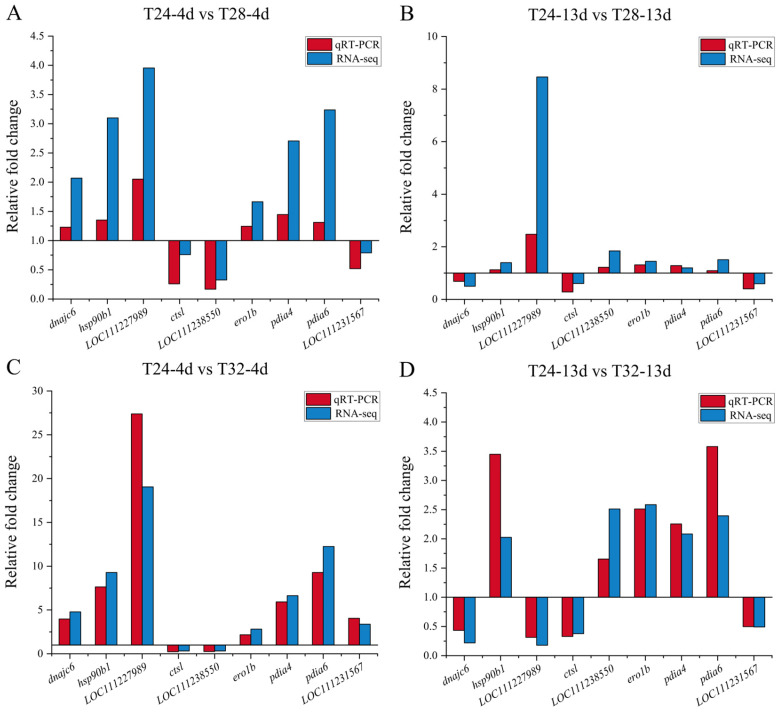
Validation of DEG expression levels using qRT-PCR. The *X*-axis shows gene names, and the *Y*-axis indicates the relative fold change between the two groups. The red bars represent the average qRT-PCR values, while the blue bars represent the average RNA-seq values. (**A–D**) Bar graphs showing the expression levels of genes in the T24-4d vs. T28-4d, T24-13d vs. T28-13d, T24-4d vs. T32-4d, and T24-13d vs. T32-13d, respectively.

The DEGs used to validate the transcriptome data included HSP genes (e.g., *dnajc6* and *hsp90b1*), oxidative stress genes (e.g., *ero1b*, *pdia4*, *pdia6*, and LOC111231567), and immune response genes (e.g., *ctsl*, LOC111227989, and LOC111238550). The results showed that the qRT-PCR data were consistent with the transcriptome analysis (Figure 5).

## 4. Discussion

### 4.1. Heat Shock Proteins’ Response to MHWs

HSP family members are key molecular chaperones involved in protein folding and biosynthesis. During cellular defense, HSPs act as important biomarkers, with their expression significantly increasing in response to heat shock [29]. In this study, 17 HSPs, including Hsp30, Hsp40, Hsp90, Hsp70, and sHSP, were significantly upregulated in *S. dumerili* under short-lasting MHWs, whereas only 3 HSPs showed significant upregulation during repeatedly occurring MHWs. Under cellular stress, denatured proteins are refolded by Hsp90 [30], which is synthesized at high temperatures to prevent cellular damage [31]. Hsp90 has been reported to play a major role in responding to sudden MHWs [32]. Hsp70 exhibits the highest sensitivity to environmental perturbations [33]. Meanwhile, Hsp40 acts as a co-chaperone to Hsp70, enhancing its ATPase activity and thereby promoting efficient protein translation, folding, and synthesis [34]. Hypoxia upregulated protein 1 (*hyou1*), a member of the Hsp70 family, which plays a crucial role in protecting cells under stress conditions [35]. This is especially important as high temperatures reduce the dissolved oxygen concentration in water. To counteract the negative effects of stressors, eukaryotic cells form networks of HSPs to prevent protein misfolding and accumulation [36]. *S. dumerili* likely forms such a regulatory HSP network in response to cellular damage, especially during short-lasting MHWs, to mitigate heat stress. Similar upregulation of HSPs in response to heat stress has been observed in other marine species, including rainbow trout (*Oncorhynchus mykiss*) (Hsp30) [37], Atlantic salmon (*Salmo salar*) (Hsp70) [38], three-spined stickleback (*Gasterosteus aculeatus*) (Hsp70 and Hsp90) [39], and Tibetan loach (*Triplophysa siluroides*) (Hsp40, Hsp70, and Hsp90) [40]. Furthermore, six Hsp40 genes were significantly upregulated under short-lasting MHWs, while two Hsp40 genes were significantly downregulated under repeatedly occurring MHWs. Molecular chaperones such as Hsp40 typically function within multi-chaperone complexes that consume ATP and use the released energy to facilitate protein folding and repair processes [41]. It is hypothesized that under MHWs, *S. dumerili* cells invest substantial energy into their protein quality control system. However, repeated MHWs may lead to energy depletion, resulting in reduced Hsp40 expression and a compensatory shift toward reliance on sHSP.

### 4.2. Oxidative Stress Under MHWs

Heat stress leads to the production and accumulation of intracellular reactive oxygen species (ROS), which disrupts ER homeostasis and induces ER stress [42]. In this study, many DEGs under short-lasting MHWs were enriched in the “protein processing in the ER” pathway, whereas only a few DEGs were enriched under repeatedly occurring MHWs. This pathway is crucial for ensuring the proper folding and degradation of misfolded proteins caused by ER stress. Genes related to the Ero1-PDI process, such as *pdia6*, *ero1a*, *ero1b*, *p4hb*, *pdia4*, and *pdia3*, were significantly upregulated under short-lasting MHWs, while *ero1b*, *pdia6*, *pdia4*, and *p4hb* were significantly upregulated under repeatedly occurring MHWs. The Ero1-PDI process promotes oxidative protein folding by releasing H_2_O_2_, thereby increasing ROS production in the ER [43]. The upregulation of the Ero1-PDI process suggests that both short-lasting and repeatedly occurring MHWs may cause protein misfolding and accumulation in the ER. Misfolded proteins are transported out of the ER lumen by binding with immunoglobulin protein through membrane channels (e.g., Derlin-1) and targeted for degradation via the ubiquitin–proteasome system [44]. Genes related to membrane channels involved in ER-associated degradation (*derl2*, *derl1*, and *tram1*) [45,46], as well as those associated with the ubiquitin ligase complex (*ube2j1*, *ube2d2*, and *ube2d4*) [31,47], were significantly upregulated in *S. dumerili* under short-lasting MHWs, indicating that the species may activate protein degradation pathways in the ER in response to short-lasting MHWs. However, no significant changes in these genes were observed under repeatedly occurring MHWs, suggesting that repeated MHW exposure may attenuate the degradation of misfolded proteins. Under short-lasting MHWs, the “protein processing in the ER” pathway also showed increased activity in proper protein folding, evidenced by the significant upregulation of molecular chaperone calreticulin genes (*calr3b*, calr3a, and *calr*), which help maintain normal protein folding and cellular function [48]. Additionally, the significant upregulation of the SEC complex gene suggests enhanced secretory functions, helping to mitigate the effects of stress on cellular pathways.

When ER stress accumulates, the unfolded protein response (UPR) activates three key signaling pathways—Ire1, PERK, and ATF6—to regulate protein synthesis and degradation inside the cell, thereby enhancing cell survival [49]. In this study, the genes associated with PERK, ATF4, IRE1, and XBP were significantly upregulated under short-lasting MHWs. Comparable upregulation has also been reported in various fish species subjected to heat stress. For example, the transmembrane proteins, PERK, IRE1, and ATF6, were upregulated in *Triplophysa siluroides* under heat stress [40]; many genes associated with the PERK-eIF2α and ATF6 pathways were upregulated in *Salmo salar* after 6 h of heat stress [50]; and high temperature induced ER stress in *Lateolabrax maculatus*, which is alleviated through the activation of the PERK/CHOP and ATF6 pathways [51]. These results suggest that two UPR pathways—PERK-eIF2α-ATF4 and IRE1-XBP1—are involved in the ER stress response triggered by MHWs, indicating that the UPR plays a protective role during cellular oxidative stress.

ER stress activates the UPR, which under severe conditions can induce apoptosis rather than cytoprotection [52]. This study showed enrichment of the apoptosis pathway, with short-lasting MHWs causing a significant increase in *caspase-6* expression. Apoptosis is regulated by caspase proteins (cysteinyl aspartate-specific proteases) that are activated by various pro-apoptotic signals [53]. During apoptosis, effector caspases such as caspase-3, caspase-6, and caspase-7 mediate cellular morphological changes, including membrane blebbing, chromatin condensation, and DNA fragmentation, by cleaving multiple apoptotic substrates, ultimately leading to cell death [54]. The crucial role of caspases in the pro-apoptotic process has also been demonstrated in heat stress studies on fish by Topal et al. [55] and Liu et al. [56]. Furthermore, the Bax-associated protein gene, a marker of apoptosis, was significantly upregulated under short-lasting MHWs. The upregulation of Bax promotes increased mitochondrial outer membrane permeability, activating the downstream caspase cascade and releasing apoptotic factors such as cytochrome c [57].

The peroxisome plays multiple roles, including maintaining redox homeostasis and facilitating the β-oxidation of fatty acids. It contains various antioxidant enzymes that help metabolize ROS [58]. Under short-lasting MHWs, the peroxisome pathway was significantly enriched, with 26 DEGs identified. Similar upregulation of antioxidant enzyme genes has been observed during heat stress in *Genypterus chilensis* and *Chanos chanos* [7,59]. The glutathione metabolic pathway was significantly enriched under both short-lasting and repeatedly occurring MHWs, with *gpx7* and *gpx8* significantly upregulated, respectively. The *gpx* genes encode glutathione peroxidase, a key antioxidant enzyme [60], suggesting that antioxidant system-associated enzymes play an active role in cellular defense during MHWs.

### 4.3. Immune Response to MWHs

Heat stress can cause fish mortality directly or indirectly by suppressing their immune system, which increases their susceptibility to pathogen invasion and disease [61]. Exposure to two MHWs led to the enrichment of immune system pathways, with most DEGs in these pathways upregulated during short-lasting MHWs but downregulated under repeatedly occurring MHWs. This downregulation of immune-related DEGs indicates impaired immunity in fish [48,62] and may contribute to the decreased survival rate of *S. dumerili* under repeatedly occurring MHW conditions. The adaptive immune system of aquatic animals such as fish is relatively underdeveloped and primarily relies on innate immunity, regulated by pattern recognition receptors such as toll-like receptors (TLRs) and NOD-like receptors (NLRs). These receptors detect damage-associated or pathogen-associated molecular patterns to initiate immune responses [63]. In this study, we observed the differential regulation of NLR and TLR signaling pathways during two MHWs. Similar patterns have been reported in previous studies on heat stress in fish [64,65]. Notably, the genes involved in the TLR and NLR pathways are generally upregulated during short-lasting MHWs but tend to be downregulated during repeatedly occurring MHWs. During short-lasting MHWs, key transcription factors such as interferon regulatory factors (IRFs), including *irf5* and *irf8*, were significantly upregulated within the TLR signaling pathway, whereas irf8 was significantly downregulated under repeatedly occurring MHWs. These factors are essential for orchestrating immune responses that target specific viral threats. Interferons (IFNs), key component in the fish immune system, are critical for regulating innate immunity and protecting against viral infections [66,67]. IRFs serve as important transcription factors that trigger IFN production. In particular, *irf5* controls the expression of type I IFN cytokines [68], while *irf8* influences both type I and type II IFNs [69]. The increased expression of *irf5* and *irf8* during short-lasting MHWs may strengthen the antiviral defenses of *S. dumerili*, enhancing its ability to resist disease. In contrast, the suppressed expression of TLR- and NLR-related genes during repeatedly occurring MHWs likely compromises the fish’s immune protection.

### 4.4. Energy Metabolism Under MHWs

To physiologically compensate for stressors, fish must mobilize energy substrates [70]. Heat stress, in particular, increases their energy demand to maintain physiological homeostasis [71]. In this study, several metabolic pathways involving amino acids, carbohydrates, and lipids were identified in both the T24-4d vs. T32-4d and T24-13d vs. T32-13d groups. These pathways included glycine, serine, and threonine metabolism; histidine metabolism; glycolysis or gluconeogenesis; and fatty acid degradation. The significant activation of these energy metabolism pathways suggests an adaptive energy allocation strategy employed by fish to cope with MHW-induced stress. Similar enrichment of these pathways under thermal stress has been reported in RNA-seq studies of various fish species [40,72,73]. Key glycolytic enzymes, hexokinase (HK) and phosphofructokinase (PFK), regulate critical early steps in the glycolytic pathway [74,75]. In this study, the genes, *hk1*, *hk2*, and *pfk1*, were significantly upregulated during short-lasting MHWs, improving the glycolytic pathway to meet the increased and fluctuating energy demands. Moreover, amino acid metabolic pathways—particularly histidine metabolism—were notably altered and enriched under these conditions. Histidine deficiency has been shown to weaken the antioxidant defenses in fish and reduce the expression of associated mRNAs [76]. Thus, the modulation of glycolysis and amino acid metabolism likely facilitates the rapid production of energy and antioxidant molecules in *S. dumerili*, ensuring the maintenance of vital physiological functions.

The pathways related to carbohydrate and amino acid metabolism were significantly enriched in response to short-lasting MHW stimuli but were not significantly affected by repeatedly occurring MHW stimuli. In contrast, the fatty acid degradation pathway was significantly altered during repeatedly occurring MHWs, a pattern also observed in *Gymnocypris chilianensis*, where heat stress caused notable enrichment of this pathway [6]. Under these conditions, fish may compensate by deriving energy from fatty acid catabolism, especially when carbohydrate metabolism is inhibited, causing cells to rely primarily on fatty acids as their energy source. This finding suggests that the metabolic capacity of *S. dumerili* to utilize energy resources for defense reduces during the transition from short-lasting to repeatedly occurring MHW stress. Previous studies indicate that as the intensity and duration of stress increase, fish may shift their strategy from “metabolic compensation” to “metabolic preservation” [77,78]. The changes in energy mobilization observed in *S. dumerili* under the two MHW events support this perspective: as the intensity, duration, and nature of the stressor increase, the energy demands of the fish may exceed their aerobic metabolic capacity, forcing a partial shift toward anaerobic metabolism to meet the energy requirements for basic life functions. This shift could push the organism into a “metabolic shutdown” state, in which all metabolic activities beyond basal metabolism are suppressed, leading to reduced carbohydrate and amino acid metabolism [78,79]. This strategic response has also been observed in other fish species under thermal stress, including olive flounder (*Paralichthys olivaceus*) [80]. Further investigation into the metabolic capacity and energy balance mechanisms of *S. dumerili* under complex MHWs stressors is needed.

## 5. Conclusions

According to transcriptome analysis, we found that *S. dumerili* maintains physiological balance under MHW stress by upregulating HSP genes (*Hsp30*, *Hsp90*, *Hsp70*, *Hsp40*, and *sHSP*), as well as *ero1b*, *pdia4*, *irf5*, *irf8*, and *hk1*. This upregulation enhances antioxidant pathways (e.g., protein processing in the endoplasmic reticulum, glutathione metabolism), immune-related pathways (e.g., TLR and NLR signaling), and energy metabolism pathways (e.g., carbohydrate and amino acid metabolism). Repeated exposure to MHWs suppresses the transcription of HSPs in *S. dumerili*, resulting in multiple functional impairments such as the breakdown of antioxidant defenses, weakened immune responses, and reduced activity in fatty acid and other energy metabolism pathways. This compromised immune function and energy metabolism likely contribute to the observed decrease in transcriptional activity and increased mortality in *S. dumerili*. These findings offer valuable insights into the molecular responses of *S. dumerili* to MHWs and can guide efforts to develop heat-resistant strains.

## Figures and Tables

**Table 1:** Sequencing and transcriptome data analysis summary.

Samples	Raw Reads	Clean Reads	Q20/%	Q30/%	Genomic Map Rate/%	GC Content%
T24-4d-1	51249386	48987888	99.56	97.45	94.76	48.5
T24-4d-2	52275924	50194262	99.6	97.54	94.20	48.5
T24-4d-3	50887318	48704476	99.58	97.59	93.90	48.5
T24-13d-1	46950386	44240394	99.47	97.26	95.21	48.5
T24-13d-2	49151540	46482634	99.51	97.44	94.14	48.0
T24-13d-3	48907528	46191450	99.55	97.51	95.65	48.5
T28-4d-1	53211760	51101020	97.53	96.03	94.99	48.5
T28-4d-2	50579226	48429602	97.36	95.75	94.18	48.5
T28-4d-3	51388726	49573704	97.24	96.47	94.64	48.5
T28-13d-1	49129624	46689354	97.45	95.03	95.59	48.5
T28-13d-2	51853392	49637240	97.35	95.73	95.17	48.5
T28-13d-3	54456140	52704094	98.08	96.78	96.69	48.5
T32-4d-1	49767488	47571478	99.56	97.48	95.49	48.5
T32-4d-2	46644588	44232310	99.47	97.37	95.43	48.5
T32-4d-3	44734602	41343752	99.45	97.34	95.27	48.5
T32-13d-1	54273686	52176884	99.64	97.75	96.87	48.5
T32-13d-2	53996890	51940710	99.78	98.01	97.11	48.5
T32-13d-3	54016228	51907586	99.71	97.79	96.83	49.0

## Data Availability

The raw sequence data have been submitted to the NCBI Short Read Archive (SRA) with accession number PRJNA1252349.

## Supplementary Materials

The following supporting information can be downloaded at: https://www.mdpi.com/article/10.3390/ani15131871/s1, Figure S1: Number of differentially expressed genes; Table S1: Primer sequence of genes for RT-qPCR; Table S2: Antioxidant, immune, and metabolism-related pathway genes in T24-4d vs. T32-4d; Table S3: Antioxidant, immune, and metabolism-related pathway genes in T24-13d vs. T32-13d; Table S4: GO term of T24-4d vs. T32-4d; Table S5: GO term of T24-13d vs. T32-13d; Table S6: KEGG enrichment pathways of T24-4d vs. T32-4d; Table S7: KEGG enrichment pathways of T24-13d vs. T32-13d; Table S8: Immune-related pathway; Table S9: Energy metabolism-related pathway.
