# Supplementary material for: Transcriptome Analyses Reveal the Molecular Response of Juvenile Greater Amberjack (Seriola dumerili) to Marine Heatwaves
Source: Animals (Basel). 2025 Jun 24;15(13):1871. doi: 10.3390/ani15131871 (PMC12249142; doi:10.3390/ani15131871)
Supplement: Supplementary file 1 [file animals-15-01871-s001.zip › Table S1.pdf]

Table S1 Primer sequence of genes for RT-qPCR

Primers	Primer sequence (5′ to 3′)
β-actin	F：TGATGAAGCCCAGAGCAAGAG<br>R：CGTTGTAGAAGGTGTGATGCCA
dnajc6	F：TGCCTACATCACATCACGG<br>R：ATTCTTGGGATTCTGTTTAAGC
hsp90b1	F：CTGGGTAAAAGCAGGGATG<br>R：GTTAGAGACAGCAAGCGGA
LOC111227989	F：ATCCCCTTAGATTGCTGCTT<br>R：TCTGAGTTGTTGGCGGTAGT
ctsl	F：CAGCCCCTACTCTGGATGC<br>R：GGACCCCTTGAACTTTCTCTCT
LOC111238550	F：TGGACGCATTGACTATGTTTG<br>R：CCAGGGAAGGAAGAAGGC
ero1b	F：GCCTGATGACGGACACTGC<br>R：GCGAACGCCTCTTTGCTC
pdia4	F：GCCCTGTTGTTGATTGTGC<br>R：TCTGTGTCATCTCCTTCGTCTT
pdia6	F：GTGGCTGATTGAGTTTTACGC<br>R：TCTGGCTTTTGCTTATTGGC
LOC111231567	F：GTCTCTGCTGCGTCGTGC<br>R：GGCTGCTGGGTGAGTTTCT
