# Supplementary figures and images for: Transcriptome Analyses Reveal the Molecular Response of Juvenile Greater Amberjack (Seriola dumerili) to Marine Heatwaves
Source: Animals (Basel). 2025 Jun 24;15(13):1871. doi: 10.3390/ani15131871 (PMC12249142; doi:10.3390/ani15131871)

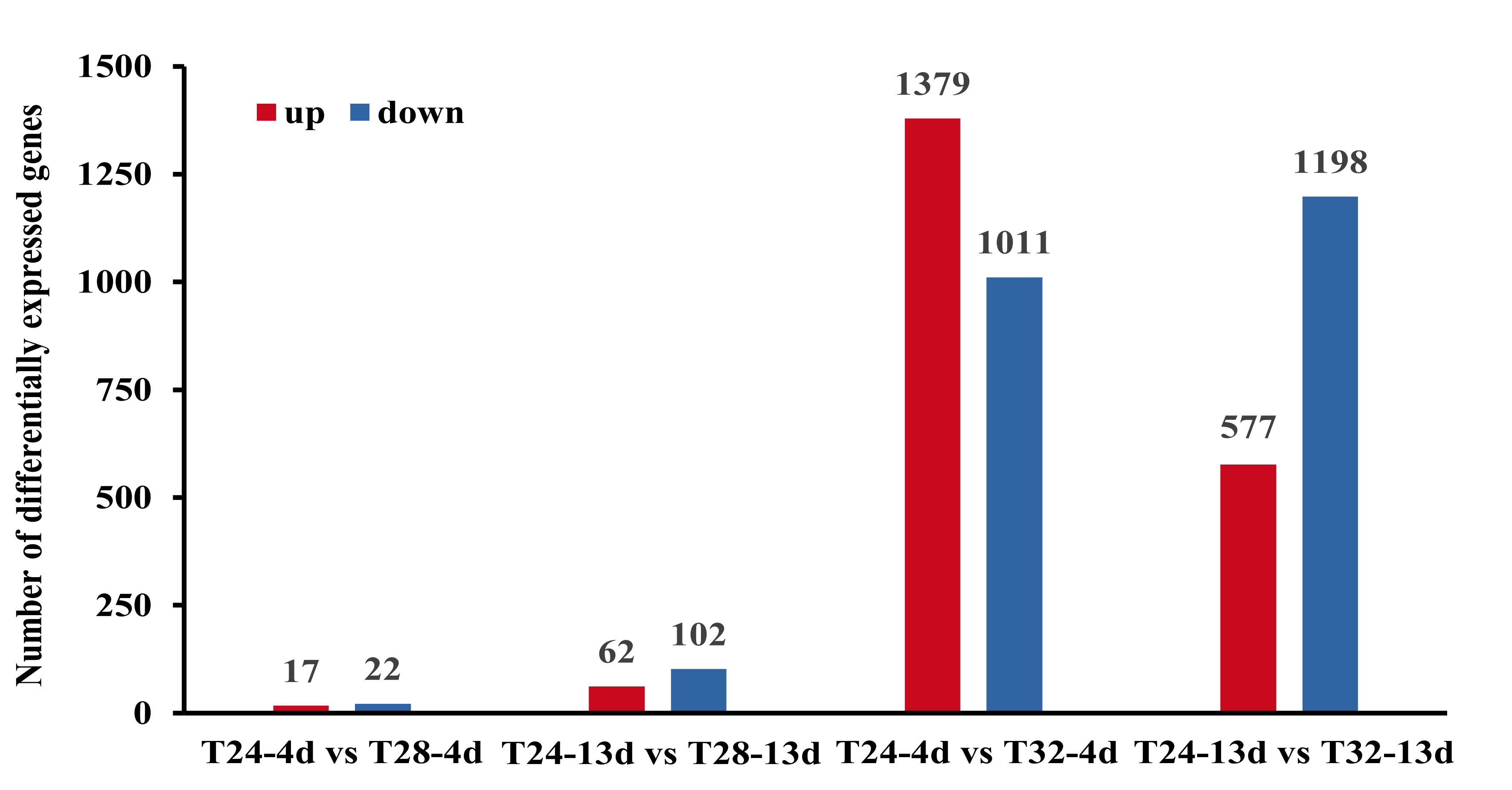

Supplement: Supplementary file 1 [file animals-15-01871-s001.zip › Figure S1.tif]
